# A Review on the Neurotoxic Effects of Doxorubicin

**DOI:** 10.1007/s12640-023-00652-5

**Published:** 2023-06-23

**Authors:** Katarzyna Kamińska, Agnieszka Cudnoch-Jędrzejewska

**Affiliations:** Chair and Department of Experimental and Clinical Physiology, Laboratory of Centre for Preclinical Research, Medical University of Warsaw, Banacha 1b, 02-097 Warsaw, Poland

**Keywords:** Chemotherapeutic drug, Central nervous system, Doxorubicin, Neurotoxicity

## Abstract

Anthracyclines, a class of drugs considered as most effective anticancer drugs, used in the various regimens of cancer chemotherapy, induce long-term impairment of mitochondrial respiration, increase reactive oxygen species, and induce other mechanisms potentially leading to neurotoxicity. According to literature findings, one drug of this class - doxorubicin used to treat e.g. breast cancer, bladder cancer, lymphoma, and acute lymphocytic leukemia may induce such effects in the nervous system. Doxorubicin has poor penetration into the brain due to the lack of drug penetration through the blood-brain barrier, thus the toxicity of this agent is the result of its peripheral action. This action is manifested by cognitive impairment and anatomical changes in the brain and peripheral nervous system found in both preclinical and clinical studies in adult patients. Furthermore, more than 50% of children with cancer are treated with anthracyclines including doxorubicin, which may affect their nervous system, and lead to lifelong damage in many areas of their life. Despite ongoing research into the side effects of this drug, the mechanism of its neurotoxicity action on the central and peripheral nervous system is still not well understood. This review aims to summarize the neurotoxic effects of doxorubicin in preclinical (in vitro and in vivo) research and in clinical studies. Furthermore, it discusses the possible mechanisms of the toxic action of this agent on the nervous system.

## Introduction

Anthracyclines are agents extracted from *Streptomyces spp* which are effective against many types of cancer (including leukemias, lymphomas, breast, stomach, or lung cancers), with a range of anti-cancer effects wider than any other class of chemotherapeutic drugs (Weiss 1992; Minotti et al. 2004). The first anthracycline discovered was daunorubicin, produced by *Streptomyces peucetius*, a species of actinobacteria. For medical purposes, the most important agents from this class are daunorubicin, doxorubicin (DOX), epirubicin, and idarubicin (Weiss 1992).

The mechanism of action of anthracyclines is based on intercalating with DNA and interfering with DNA metabolism and RNA production. Intercalating is the phenomenon of binding of small molecules inside the molecules of macromolecular compounds or inside supramolecular structures made of molecules bound to each other by, for example, hydrogen bonds or van der Waals interactions. Like other anti-cancers agents administration of anthracyclines is often accompanied by adverse drug reactions e.g. nausea and vomiting, skin and nail hyperpigmentation, photosensitivity, and neutropenia but their main adverse effect is cardiotoxicity, which considerably limits their usefulness (Douedi and Carson 2021). This cytotoxicity is primarily due to the inhibition of topoisomerase II, an enzyme that cuts both strands of the DNA helix simultaneously, preventing relegation of the break and leading to cell death.

Anthracyclines may also have nonspecific effects in the field of neurotoxicity, which may manifest clinically with cognitive impairment, and is associated with anatomical changes in the brain and peripheral nervous system found in both preclinical and clinical studies (Kesler and Blayney 2016; Lim et al. 2016).

Considering the facts, that:

- depressive syndromes occur in about 20-40% of cancer patients, and in a third of those cases are present at the time of confirmation of a cancer diagnosis,
- the percentage of depressive states decreases to 15% in cancer patients in remission, only to rise again to as high as 50% at cancer recurrence, 

(https://www.zwrotnikraka.pl/depresja-w-chorobie-nowotworowej/ Accessed 10 June 2022) the understanding of possible neurotoxic effects and mechanisms of action of chemotherapeutics is needed, especially if their administration can exacerbate depressive episodes by, among others, worsening cognitive impairment. It was found that chemotherapy-related cognitive impairment is estimated to occur in 17–75% of patients receiving cancer chemotherapy. Moreover, 17–30% of the afected patients appear to sustain long-term cognitive impairment after chemotherapy (El-Agamy et al. 2019).

It is also worth mentioning that more than 50% of children with different types of cancer are treated with anthracyclines, which may affect their nervous system (Armenian and Bhatia 2018). For example, pediatric patients with acute lymphoblastic leukemia (ALL) receiving chemotherapy demonstrate a decrease in the hippocampus (HP), amygdala (AMY), thalamus (TH), and nucleus accumbens (NAc) gray matter volume, as well as loss of white matter in the HP and TH. Furthermore, a lower ratio of white matter to intracranial volume in the frontal and temporal lobes was found in pediatric cancer patients (Marusak et al. 2018). These abnormalities manifest in signs of cognitive impairment and can include difficulty in paying attention and taking an extremely long time to perform tasks, such as homework. In comparison with healthy controls (siblings) pediatric cancer patients after chemotherapy were three to four times more likely to have learning disabilities and deficits in social skills compared with other children their age (Haupt et al. 1994). Thus, the neurotoxic effects of anthracycline treatment have the potential to cause lifelong damage in many areas of their life.

One of the anthracycline drugs that may cause neurotoxicity is DOX, used to treat e.g. breast cancer, bladder cancer, lymphoma, and acute lymphocytic leukemia.

## Doxorubicin

**Fig. 1:**
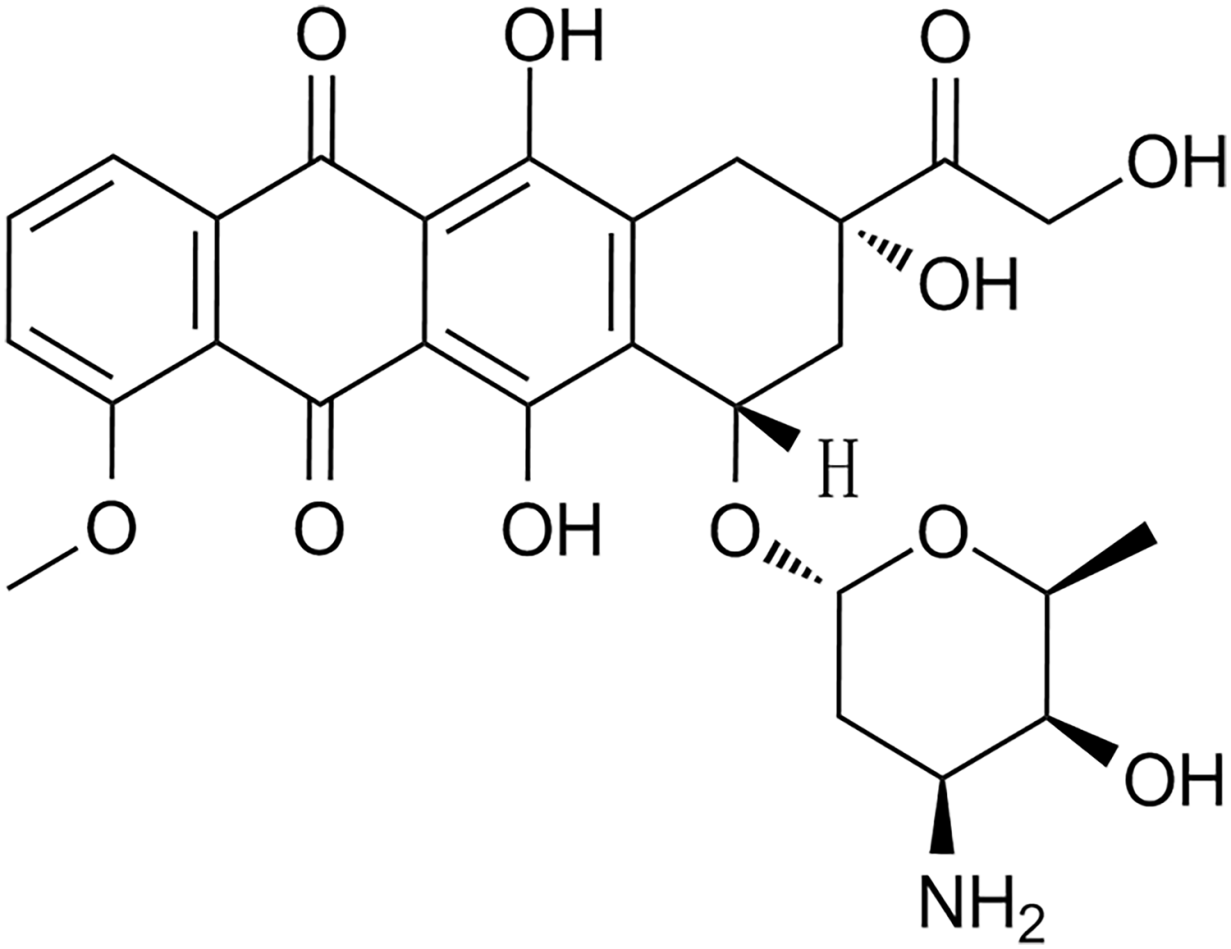
Chemical structure of DOX ((7S,9S)-7-((2R,4S,5S,6S)-4-amino-5-hydroxy-6-methyloxan-2-yl) oxy-6,9,11-trihydroxy-9-(2-hydroxyacetyl)-4-methoxy-8,10-dihydro-7H-tetracene-5,12-dione)

DOX ((7S,9S)-7-((2R,4S,5S,6S)-4-amino-5-hydroxy-6-methyloxan-2-yl)oxy-6,9,11-trihydroxy-9-(2-hydroxyacetyl)-4-methoxy-8,10-dihydro-7H-tetracene-5,12-dione), a deoxy hexoside anthracycline, was isolated from *Streptomyces peucetius var. caesius* bacteria*.* DOX is a member of p-quinones, a primary alpha-hydroxy ketone, and a tertiary alpha-hydroxy ketone (https://pubchem.ncbi.nlm.nih.gov/compound/Doxorubicin. 03.03.2022). The chemical structure of DOX is shown in the Fig. 1.

This agent has three metabolic pathways: one-electron reduction, two-electron reduction facilitated by several oxidoreductases to form a DOX-semiquinone radical, and deglycosylation considered as a minor metabolic pathway (1-2% of the dose undergoes this pathway). Metabolites of this pathway are deoxyaglycone or hydroxyaglycone formed by either reduction or hydrolysis, respectively (https://www.pharmgkb.org/pathway/PA165292177, Accessed 01.06.2022).

The principal mechanism of action of DOX is still not fully understood but is related to DNA intercalation and inhibition of macromolecular biosynthesis (Fornari et al. 1994; Tacar et al. 2013). This inhibits the progression of topoisomerase II, an enzyme that relaxes supercoils in DNA, thus facilitating the transcription process (Pommier et al. 2010). DOX stabilizes the topoisomerase II complex after the DNA chain is broken for replication, preventing the release of the DNA double helix and thus stopping the replication process (Tacar et al. 2013). It can also increase the production of quinone-type free radicals (FR), thus contributing to its cytotoxicity (Rossi 2013). The flat aromatic chromophore portion of the molecule intercalates between the two DNA base pairs, while the daunosamine six-membered sugar is located in the smaller groove and interacts with the flanking base pairs immediately adjacent to the intercalation site as evidenced by several crystal structures (Pigram et al. 1972; Frederick et al. 1990). DOX is also able to induce the eviction of histones from transcriptionally active chromatin via intercalation (Pang et al. 2013).

As a result, DNA damage response, epigenome, and transcriptome are deregulated in the cells exposed to DOX action (Pang et al. 2013). The cytotoxic effect of DOX results from a complex system of multiple modes of action related to FR and reactive oxygen species (ROS) formation, intercalation of the drug into DNA, induction of DNA breaks and chromosomal aberrations, and alterations in cell membranes. In vitro studies in cells treated with DOX suggest that apoptosis also may be involved in the drug's mechanism of action (Tomankova et al. 2015, Pilco-Ferreto and Calaf 2016).

Like the other anthracyclines, DOX has a lot of adverse effects. Patients respond differently to chemotherapy. Some experience few side effects, while others experience more. The most common symptoms of DOX adverse effects include nausea, vomiting, stomatitis, loss of appetite, stomach pain, diarrhea, increased thirst, unusual tiredness or weakness, dizziness, hair loss, separation of fingernail or toenail from the nail bed, ocular pain or red discoloration of urine. However, the progressive cardiotoxicity usually occurring after the completion of treatment with anthracyclines limits the use of DOX (Simůnek et al. 2009). Dilated cardiomyopathy, leading to congestive heart failure is the most dangerous side effect of it (Chaterjee et. al. 2010). DOX cardiotoxicity can be acute, with a prevalence of about 11%, and chronic, with an estimated incidence of about 1.7%. Acute cardiotoxicity occurs within the first 2–3 days of DOX administration, while chronic cardiotoxicity usually manifests clinically within 30 days of administration of its last dose, but it may occur even after 6–10 years after its completion (Von Hoff et al. 1979; Takemura and Fujiwara 2007). The acute cardiomyopathy induced by DOX is usually manifested through chest pain due to myopericarditis and/or palpitations due to sinus tachycardia, paroxysmal nonsustained supraventricular tachycardia, and premature atrial and ventricular beats. The incidence of DOX-induced cardiomyopathy is related to its dose. It is about 4% when the dose is 500–550 mg/m2, 18% when 551–600 mg/m2, and 36% when 600 mg/m2.

Furthermore, more and more literature reports in the field of basic and clinical research indicate that DOX exposure may induce neurotoxicity, especially in synaptic processes associated with hippocampal neurotransmission (Alhowail et al. 2019). This aspect of DOX's negative effects has not yet been well-studied.

It is known that DOX has a weak ability to penetrate through the BBB. At this point, it is worth mentioning that the mechanism of BBB-mediated drug resistance is complicated by the interaction of P-glycoprotein (P-gp, ABCB1) and breast cancer resistance protein (BCRP, ABCG2), which is successful in removing molecules and drugs from the CNS (Löscher and Potschka 2005). Despite those facts in vitro and in vivo data on models of malignant glioma suggest that this drug is an effective anti-tumor agent (Liang et al. 1991; Muldonn and Neuwelt 2003).

## Neurotoxicity of Doxorubicin

It was believed that DOX is completely unable to cross the BBB, however, many preclinical studies on animal models reported neurotoxicity associated with its administration in various doses (Mohamed et al. 2011; Rizk et al. 2017; Liao et al. 2018). Moreover, research on animals has also shown that DOX low levels were detected in the brain after intraperitoneal (*ip*) administration (Sardi et al. 2013). Currently, research is being conducted to increase the availability of DOX in the brain to treat cancers that occur there. However, this may exacerbate its neurotoxicity.

Later, it was suggested that DOX can cross the BBB through vascular-associated apical projections of neural stem cells. Thus, it can establish direct membrane-membrane contacts with the endothelial cells in specific regions of the irregular endothelial basement membrane, and have abundant vesicular activity (Licht et al. 2020; Du et al. 2021).

## In Vitro Studies

Research conducted by Lopes (2008) proved that DOX is neurotoxic to serum-free cultures of cortical neurons of Wistar rats – the primary cultures of cerebral cortex obtained from embryos (E17-18). DOX concentrations up to 0.5 μM, induced cell death through an apoptotic pattern, while for higher concentrations (5, 10, 20 µM) necrosis becomes dominant (Lopes et al. 2008).

A study by Petrovic et al., showed that DOX (1μM for 17 h) affects the expression of proteins of pathways related to neuronal development (CNS neuron differentiation, neuron projection membrane, soluble Soluble N-ethylmaleimide-Sensitive Factor Attachment Proteins attachment proteins receptor activity) of the MCF-7 breast cancer cell line, using the precursor acquisition independent from ion count mass spectrometry method (Petrovic et al. 2015). This research found that proteins like myotrophin, mitochondrial 2- oxoglutarate dehydrogenase, eukaryotic translation initiation factor3 subunit, robable E3 ubiquitin-protein ligase microtubule-associated protein 2 involved in the above-mentioned pathways, which are crucial to physiological processes in the central nervous system, are down-regulated when exposed to treatment with DOX. These findings might explain the development of cognitive impairment symptoms which occur after chemotherapy in cancer patients (Petrovic et al. 2015).

The experiment performed by Ramalingayya et al. (2017a) demonstrated that IMR32 cells exposed to DOX (1µM for 24 h) exhibit increased apoptosis, and intracellular ROS generation with simultaneous inhibition of neurite growth (Ramalingayya et al. 2017a).

In another research, a hippocampal cell line (H19-7/IGF-IR) along with rodent hippocampal slices was tested to evaluate the acute neurotoxic effects of DOX at a concentration 0.25, 0.5, 0.75, and 1 µM (Alhowail et al. 2019). The reduction in long-term potentiation (LTP) in hippocampal slices with DOX was observed. In addition, the markers of oxidative stress - lipid peroxidation and caspase-3 expression were increased in investigative cells at a long site with extracellular signal-regulated kinase 1/2 (ERK1/2), p38 mitogen-activated protein kinase, and Akt (Gururaj et al. 2005, Alhowail et al. 2019).

A series of studies by Jantas and co-workers, who investigated cell death evoked by DOX in human neuroblastoma SH-SY5Y cells (Jantas et al. 2008; 2015; 2018a; Chwastek et al. 2017), showed that DOX at the concentration of 0.5 μM caused the apoptotic fragmentation of DNA in undifferentiated -SHSY5Y cells (UN-SHSY5Y) without being harmful to retinoic acid-differentiated -SHSY5Y cells (RA- SHSY5Y) (Jantas et al. 2008). It was also proved that 1 μM of DOX produced necrotic changes in undifferentiated -SHSY5Y and evoked apoptosis in retinoic acid-differentiated -SHSY5Y (Jantas et al. 2008). Furthermore, the group investigated the cell-damaging effect of DOX in the primary cortical, hippocampal and striatal neurons (Jantas and Lasoń 2009). The data revealed that cerebellar neurons were the most resistant to DOX-induced apoptosis when compared to neuronal cell cultures derived from the forebrain (Jantas and Lasoń 2009). Moreover, programmed cell death induced by DOX in a concentration-dependent manner had a higher damaging effect in immature neurons (Jantas and Lasoń 2009). Jantas and co-workers (2018b) proved also that DOX is neurotoxic for cortical glia cell cultures (Jantas et al. 2018b).

The results of in vitro data showing the DOX-induced neurotoxicity are presented below in the Table 1.

**Table 1:** Summary of in vitro data

**Cell line**	**Dose**	**Effect**	**Ref.**
neurons of cerebral cortex from embryos (E17-18) of Wistar rats	0.5µM	death via apoptosis	Lopes et al. 2008
	5, 10, 20 µM	death via necrosis	
MCF-7 breast cancer cell line			
Human neuroblastoma (IMR32) cells	1μM for 17 h	proteins down regulation	Petrovic et al. 2015
hippocampal cell line H19-7/IGF-I			
	1µM for 24 h	↑ apoptosis	Ramalingayya et al. 2017a
		↑intracellular ROS	
human neuroblastoma SH-SY5Y UN- and RA-SH-SY5Y cells	0.25, 0.5,	Reduction in LTP in hipocampus	Alhowail et al. 2019
	0.75, 1µM	↑ of oxidative stress markers	
human neuroblastoma SH-SY5Y UN- and RA-SH-SY5Y cells			
		0.5 μM caused the apoptotic fragmentation of DNA	
	0.1–5 μM for 6, 14 and 24 h	1 μM necrotic changes	Jantas et al. 2008
human neuroblastoma SH-SY5Y			
		Dox effect on caspase-3 12 h treatment was employed	
	Dox: 0.25 and 1 µM for UN- and RA-SH-SY5Y cells for 6 and 12 h		
			Jantas et al. 2015
	0.1–1 μM for 24 and 48h		
cortical glia cells from 2-day-old Albino Swiss or C57Bl/6J mice		0.1–0.5 μM a time-dependent decrease in cell viability in UN-SH-SY5Y	
		0.1–1 μM RA-SH-SY5Y cell damage	
			Jantas et al. 2018a
human neuroblastoma SH-SY5Y	1 μM for 48h and	a significant increase (50 %) in caspase-3 activity by 0.1 µM AMN082 when it was given with Dox	
	0.25-2 µM for 24 and 48 h (cell response to various harmful agents)		
	0.25 and 1 μM for UN- and RA-SH-SY5Y cells for 24 h	significantly reduced cell viability	
primary cultures of mouse hippocampal, neocortical, striatal, and cerebellar neurons			
	0.1–5 μM for 6, 14, 24 and 48 h		
			Jantas et al. 2018b
		cell death with higher damaging effect in immature neurons	
			Chwastek et al. 2017
			Jantas 2009

### Animal Studies

#### Behavioral tests

In the study of Liedke et al. (2009) the effect of a single dose of DOX 8 mg/kg (equivalent to the human dose of 60 mg/m2) on memory for inhibitory avoidance conditioning in 2 to 3 months old Wistar rats was investigated. The experiments showed a decrease in exploratory behavior assessed by the number of rearings during the exploration of an open field in DOX-treated rats. The results indicate that an exposition to a systemic administration of DOX might impair long-term learning (Liedke et al. 2009).

In another study, it was presented that rats (strain not specified in the cited article) exposed to DOX action (4 mg/kg/week for 4 weeks, *ip*) exhibited a significant decrease in the number of arm entries, and spontaneous alternation percentage compared to control in the Y-maze test, which suggests the reduction in short-term and long-term memory (Alharbi et al. 2020). Additionally, 6-week-old athymic (T-cell deficient, partially immunocompromised) male mice (NCr nude) treated with DOX (5 mg/kg for 5 weeks, *ip*) demonstrated impaired performance in the Y-maze and a significant reduction in the hippocampal long-term potentiation (Alhowail et al. 2019).

According to Ramalingayya and co-workers' study, animals treated with DOX (2.5 mg/kg, i.p., every 5 days for 50 days) showed an insignificant difference in exploration time of the novel or familiar object in comparison to the control group in the novel object recognition test (NOR) (Ramalingayya et al. 2017b). In the Moretti et al. study (2021) DOX was administered (2.5 mg/kg/week for 4 weeks, *ip*) to male rats (Wistar), which showed short-term and long-term memory impairments in NOR test at 3 and 24 h after habituation (Moretti et al. 2021). Furthermore, this study suggests that DOX induces hippocampal gene expression changes, which is related to an increase in anxiety behavior in young animals (Moretti et al. 2021). These studies are in line with previous research which documented increased anxiety and impaired spatial cognition in rodents after DOX injection (2- 2.5 mg/kg for 4 weeks, *ip*) (Kitamura et al. 2015; Philpot et al. 2016). What is interesting, the DOX-induced changes in cognitive behavior may be more severe in female than in male animals (Cavalier et al. 2021). In contrast, in the studies of Aziriova et al. (2014) and Kitamura et al. (2015), no anxiety-like behavior was statistically demonstrated after DOX administration (Aziriova et al. 2014, Kitamura et al. 2015). Additionally, no differences were reported after DOX administration in the open field test where locomotion and rearing frequencies were measured during the 4-week-long experiment (El-Agamy et al. 2018).

In research (Merker et al. 1978) on rhesus monkeys, DOX was perfused through the ventriculo-cisternal and ventriculo-lumbar spaces at concentrations from 1.5 to 100 μg/ml for 190 min. The neurotoxicity after perfusion manifested in body weakness, tremors, severe to slight hypokinesia, excitation, nervousness, or depression. In the brains of three monkeys a distinctive necrotizing angiopathy that was noninflammatory was found (Merker et al. 1978).

On the contrary, in the study by Flanigan et al. (2018) where DOX (2 mg/kg) and cyclophosphamide were administrated to C57BL/6J mice, only sporadic effects due to chemotherapeutic treatment were observed (Flanigan et al. 2018).

The results of various studies reflecting DOX-induced neurotoxicity are presented in Table 2.

**Table 2:** Summary of behavioral, biochemical, and histopathological data

**BEHAVIORAL**				
**Experiment**	**Dose**	**Species/ Strain**	**Effect**	**Ref.**
Avoidance	8 mg/kg, *ip*	rat/Wistar	↓ exploratory behavior	Liedke et al. 2009
conditioning				
			short-term and long-term memory reduction	Alharbi et al. 2020
Y-maze	4 mg/kg x 4 weeks, *ip*	rat/not specified		
			reduction in hippocampal long-term potentiation	
				Alhowail et al. 2019
			insignificant difference in exploration time of the novel or familiar object	
Y-maze	5 mg/kg x 5 weeks, *ip*	mice/NCrnude		
			short-term and long-term memory impairments	Ramalingayya et al. 2017a
			↑ anxiety and impaired spatial cognition	
NOR	2.5 mg/kg, ip, every 5 days for 50 days, *ip*	rat/Wistar		
			deficits in spatial memory	Moretti et al. 2021
			no anxiety-like behavior	
	2.5 mg/kg x 4 weeks, *ip*			Kitamura et al. 2015
NOR		rat/Wistar		
				Philpot et al. 2016
	2 mg/kg, *ip*		no anxiety-like behavior	
Locomotor		rat/Wistar		Aziriova et al. 2014
activity				
	2.5 mg/kg, *iv*		no differences	
Morris		mice/BALB/		
Water Maze				Kitamura et al. 2015
	5 mg/kg, *ip*			
Open field		rat/Wistar	weakness, tremors, severe to slight hypokinesia, excitation, nervousness, depression	
Elevated plus maze				
The light/dark box				El-Agamy et al. 2018
Locomotor activity				
Anhedonia like behavior	2 mg/kg,* ip*			Merker et al. 1978
		rat/Wistar		
open field test				
	2 mg/kg x 4 weeks, *ip*			
Behavior observation		rat/Albino		
	1.5 to 100 μg/ml for 190 min, *ip*			
		monkey/Rhesus		
**BIOCHEMICAL**				
**Tissue**	**Dose**	**Species/ Strain**	**Effect**	**Ref.**
all brain	20 mg/kg, *ip*	mice/ not specified	↑ lipid peroxidation	Joshi et al. 2005
			↑ protein oxidation	
			↑ MRPI	
				Tangpong et al. 2006
all brain	20 mg/kg*, ip*	mice/ B6C3	↑ p53 and Bax 3 & 72 h after DOX inj.	
			↑ Bcl-xL 6 h after DOX inj.	
			↑ MDA	Mohammed et al. 2019
			↑ Total protein level	
all brain	10-20 mg/kg,* ip*	rat/Wistar Albino		
				Kuzu et al. 2018
			↑ TNF-α	
				Imosemi et al. 2022
all brain	40 mg/kg, *ip*	rat/Wistar Albino		
			↑ TNF-α	
cerebrum, hypothalamus, cerebellum	2 mg/kg,* ip*	rat/Wistar Albino		
**HISTOPATOLOGICAL**				
**Structure**	**Dose**	**Species/ Strain**	**Effect**	**Ref.**
all brain	10 mg/kg,* ip*	rat/not specified	degeneration in high amount of neurons	Eddy and Nathaniel 1982
				Tangpong et al. 2006
			↑ TNF-α	
all brain	20 mg/kg,* ip*	mice/ B6C3	↑apoptotic cells	Manal et al. 2018
			neurotoxicity	Leung et al. 2020
all brain	2.5 mg/kg every other day for 2 weeks,* ip*	rat/not specified		
				Moretti et al. 2021
	3.5 mg/kg/week for 8 weeks		hioppocampus degradation	
hipocampus		rat/Wistar		
	2.5 mg/kg/week for 4 weeks, *ip*		↑GFAP	
			↑AIF-1	
Frontal cortex, hypothalamus, hipocampus		rat/Wistar		

#### Biochemical tests

Joshi and co-workers (2005) demonstrated a significant increase in levels of lipid peroxidation, protein oxidation, and increased expression of multidrug resistance-associated protein (MRP1) in the brain isolated from mice, 72 h post *ip* injection of DOX (20 mg/kg) (Joshi et al. 2005).

The other study revealed that DOX produced a significant increase in IL-1β levels in all of the brain tissue homogenates in comparison to the control (Tangpong et al. 2006). In the same experiment, the levels of pro-apoptotic proteins, p53 and Bax, were measured in the brain-derived mitochondria 3 h after DOX administration. The increase in both proteins level was noted. Interestingly 6 h after DOX injection Bcl-xL – the anti-apoptotic protein level was increased (Tangpong et al. 2006).

The research by Mohammed et al. (2019) showed that *ip* administration of 4 mg/kg of DOX (once a week for four weeks on days 7, 14, 21, and 28) caused a significant reduction in the relative cerebellum, cerebrum, and hypothalamus weight in Wistar albino rats. Moreover, the increase in the levels of TNF-α and malondialdehyde (MDA), and the total protein level in the brain tissue of the DOX-treated group compared to the control group was observed (Mohammed et al. 2019). In contrast, reduced glutathione (GSH) and glutathione peroxidase (GPx) levels were decreased.

A similar observation was made by Kuzu et al. (2018) who reported a significant increase in TNF-α levels in brain tissues of Wistar albino rats after DOX administration (40 mg/kg) (Kuzu et al. 2018).

In the work of Imosemi et al. (2022) the effect of an antioxidant - luteolin (50 or 100 mg/kg; per os) on DOX (2 mg/kg; *ip*)- induced oxidative stress, inflammation, and apoptosis was investigated in the brain of Wistar rats (Imosemi et al. 2022). The results showed that luteolin reduced DOX-mediated markers of oxidative stress, such as catalase, superoxide dismutase (SOD), glutathione-S-transferase (GSTs), and glutathione peroxidase (GPx). The DOX-treated rats also showed increased levels of acetylcholinesterase in the cerebellum, cerebrum, and hypothalamus, which may lead to harmful neurological effects. Luteolin elevated the level of glutathione. Furthermore, it alleviated DOX-induced increase in the levels of lipid peroxidation, myeloperoxidase, nitric oxide (NO), TNF-α, and interleukin-1β (IL-1β), thus decreasing the DOX-induced neurotoxicity (Imosemi et al. 2022).

The results of biochemical studies which reflect DOX-induced neurotoxicity are presented in Table 2.

#### Histopathological Examination

The neurotoxic effects induced by DOX were also confirmed by the histopathological examination:

Electron microscopic observations of DOX (10mg/kg) treated young and adult rats showed various stages of degeneration in a high number of neurons with the most severe changes occurring in the 1-week-old animals. The neuronal changes during the neurodegenerative process were characterized by dilated cisternae peripherally and elevated bundles of filaments causing irregular dispersal of organelles. Moreover, progressive changes were noted with the accumulation of vesicular profiles, membranous figures, and electron-dense bodies in the neurons. Similar accumulation was seen in myelinated axons, while the myelin sheath appeared relatively intact (Eddy and Nathaniel 1982).

The confocal microscopy analysis made by Tangpong and co-workers (2006) demonstrated that TNF-α levels increased in neurons of B6C3 mice 3 h after injection of 20 mg/kg DOX compared to the control (Tangpong et al. 2006). Furthermore, 3 and 72 h after DOX treatment, an increased number of apoptotic cells was found in both cortical and hippocampal cells using the TUNEL method (Tangpong et al. 2006).

Ramalingayya and co-workers revealed that cerebral cortex samples of the Wistar rats treated with DOX showed degenerative changes as compared to the control. Moreover, the same experiment showed that co-treatment with DOX along with rutin at a dose of 50 mg/kg retains pathological changes observed with DOX administration alone (Ramalingayya et al. 2017b).

Neuronal degeneration in the rat brain and with frequent nuclear pyknosis was observed in Manal et al. (2018) study, after *ip* administration of DOX at a dose of 2.5mg/kg. Histologically it was characterized by irregular darkly stained cells with pyknotic nuclei, surrounded with halos (Manal et al. 2018).

In the study by Leung et al. (2020), the degeneration of cellular constituents of HP was found in DOX-exposed (3.5 mg/kg/week for 8 weeks) rats (Leung et al. 2020).

Moretttii et al. (2021) in their study showed an increased glial fibrillary acidic protein (GFAP) and allograft inflammatory factor 1 (AIF-1) also known as ionized calcium-binding adapter molecule 1 (IBA1) expression, in astrocytes and microglia of the Wistar rat frontal cortex, hypothalamus and HP after DOX administration (2.5 mg/kg/week for 4 weeks) (Moretti et al. 2021).

The results of histopathological studies reflecting the DOX-induced neurotoxicity are presented in Table 2.

### Clinical Studies

The neurotoxicity of DOX in humans is characterized by various symptoms, including cognitive dysfunction like memory loss, the tendency for distractions, difficulty in performing multiple tasks, and depressive episodes (Yang 2013). The aforementioned symptoms have been reported in cancer patients, especially breast cancer, undergoing DOX-based chemotherapy (Freeman and Broshek 2002). Moreover, a lot of neurological symptoms like the presence of a headache, seizures, encephalopathy, and visual disturbances was observed (Mo’mena et al. 2021; Khan et al. 2016; Kamiya-Matsuoka et al. 2016).

In a 16-year-old male patient diagnosed with acute lymphoblastic leukemia subtype B chemotherapy with methotrexate, DOX and cyclophosphamide were started. The patient reported a headache and decreased strength in the right arm and aphasia (Lederman et al. 2017).

In the study of Mo’mena and co-workers (2021) 36 patients diagnosed with leukemia, 24 with lymphoma, and 3 with solid malignancies were receiving chemotherapy treatment: vincristine, methotrexate, corticosteroids, Ara C and DOX. All patients were aged 1–14 years. All of the investigated children developed different neurological syndromes with the most common being the posterior reversible encephalopathy syndrome (PRES), which seizures as the most common presenting symptom (Mo’mena et al. 2021). These results are in agreement with the study of Khan et al. (2016) in which 37 children’s patients were diagnosed with PRES (Khan et al. 2016). Furthermore, in Kamiya-Matsuoka et al.’s research (2016) 69 cancer patients with the aforementioned syndrome were identified, and in 70% of them, the most commonly administered agents were DOX and vincristine (Kamiya-Matsuoka et al. 2016). PRES is a clinico-radiologic entity, and it became an increasingly recognized condition in cancer patients undergoing chemotherapy, the main symptoms of which are headache, seizures, encephalopathy, and visual disturbances (Khan et al. 2016).

In the meta-analysis performed by Eide and Feng (2020) 511 DOX-treated women with breast cancer was compared to 306 healthy women across measures of defined cognitive modalities. DOX-treated patients experience significant impairment in global cognition in comparision to the controls. It was found that within memory, short-term verbal memory was most significantly affected. Impairment in select cognitive modalities (executive function, language, memory, short-term verbal memory, processing speed) was prevalent in DOX-treated patients, with some cognitive functions remaining intact (Eide and Feng 2020).

The results of clinical studies reflected the DOX-induced neurotoxicity are presented in Table 3.

**Table 3:** Summary of clinical studies

**Patients**	**Symptomes**	**Ref.**
breast cancer patients	cognitive impairment	
17 women with breast	cognitive impairment	Freeman and Broshek 2002
cance		
16 yo male	headache and decreased strength	Lederman et al. 2017
	in right arm and aphasia	
36 patients	neurological syndromes in all, most common PRES and seizures	Mo’mena et al. 2021
1-14 yo		
	PRES	
37 children’s patients		Khan et al. 2016
	PRES in 70% patients	
69 cancer patients		Kamiya-Matsuoka et al. 2016

## DOX Neurotoxicity in the Peripheral Nervous System

There is very little literature data on the toxicity of DOX on the peripheral nervous system.

In a study by Cho (1977) the neurotoxicity of DOX administrated in a dose of 10 mg/kg intravenously (*iv*) in rats (strain not specified in the cited article) has been demonstrated by changes in ganglion cells of the peripheral nervous system with sparing of neurons in the CNS with the light microscopy. Necrosis of neurons was followed by mild lymphocytic infiltration, and ultimate loss of ganglion cells in the spinal, paravertebral and trigeminal ganglia. Moreover, the elevated numbers of neurofilaments and the presence of membrane-bound cisterns in the affected dorsal root ganglion cell bodies were revealed by electron microscopy (Cho et al. 1977).

Other research performed on Sprague-Dawley-rats exposed to DOX (10 mg/kg) action showed neuronal necrosis in peripheral ganglia which was most developed in the dorsal root ganglia (Cho et al. 1980). The DOX effect on the ganglion neurons was manifested in the nuclei with focal clearing (3 hours after drug administration) and disintegration of chromatin material (by 4 days). This was followed by morphological changes in the bodies of neurons, accompanied by Wallerian degeneration of the associated nerve fibers. According to the authors, these changes occurred as a result of the fact that DOX was able to pre-sot through porous blood vessels in the dorsal root ganglia and reach neuronal nuclei and stellate cells soon after administration (Cho et al. 1980).

It was also found that DOX elicits ganglioneuropathy in rabbits (Bronson et at. 1982). Rabbits administrated with 12 mg/kg of DOX had only mild degenerative changes in dorsal roots and a few necrotic neurons in the dorsal root ganglia. Injections with 16 mg/kg had much more severe effects (Bronson et al. 1982). The ganglioneuropathy was also induced in a similar pattern of experiment in a rhesus monkey after receiving a total of 20 mg/ kg DOX over a 10-month period (Bronson et al. 1982).

Quantitative analysis of neuronal counts of the ganglia of rats administrated with 10 mg/kg of DOX showed a significant loss of sensory neurons. Furthermore, the remaining neurons had many morphological abnormalities which suggested a degenerative process (Eddy 1983).

The results of studies on the peripheral nervous system reflected the DOX-induced neurotoxicity are presented in Table 4.

**Table 4:** Summary of research on neurotoxicity in the peripheral nervous system

**Structure**	**Dose**	**Species/ Strain**	**Effect**	**Ref.**
ganglion cells in the spinal, paravertebral and trigeminal ganglia	10 mg/kg	rat /not specified	necrosis of neurons	Cho et al. 1977
peripheral ganglia, spinal cord neurons				
				Cho et al. 1980
dorsal roots and ganglia	10 mg/kg	Sprague-Dawley-rats	morphological changes in neurons/ degeneration of the associated nerve fibers	
			degenerative chang-es in rabbits/ gan-glioneuropathy in monkeys	
ganglia			loss of sensory neurons	Bronson et al. 1982
	12-16 mg/kg rats	rabbits and rhesus monkey		
	20 mg/kg monkeys			
				Eddy 1983
		rat/not specified		
	10 mg/kg/wt			

## The Mechanism of Neurotoxicity

DOX can induce cell death via different mechanisms depending on its concentration. In lower doses, DOX induces mainly programmed cell death by the extracellular (FAS - death receptor on the surface of cells) pathway of apoptosis with activation of caspase-8 activity (Bian et al. 2001; Jantas et al. 2008). This step requires the activation of certain transcription factors and the production of proapoptotic factors (Bian et al. 2001). Furthermore, a high concentration of DOX can induce DNA damage, overproduction of ROS, and depolarization of the neuronal mitochondrial membrane (Ren et al. 2019). Manchon and co-workers proved that DOX accumulated in the neuronal nuclei, leading to DNA double-strand breaks and DNA cross-linking (Di Bartolomeo et al. 2000). DOX increased the mitochondrial outer membrane permeability (MOMP) and two cytoplasmic proteins Bax/Bcl-2 ratio, facilitated the release of cytochrome C, and activated the caspase-dependent apoptotic pathways (Shokoohinia et al. 2014; Lee and Lee 2018).

Due to their increased metabolism, neurons are more susceptible to oxidative damage than other cells. Oxidative stress and neuron degeneration caused by abnormal mitochondria activation are important causes of cognitive impairment affecting learning and memory (Ramalingayya et al. 2017a). Exposure to DOX may cause neuronal damage by promoting oxidative stress and increasing the plasma levels of TNF-α, which can easily pass the BBB (Tangpong et al. 2006). Furthermore, TNF-α can activate the microglia in the brain, thus elevating the production of NO which leads to mitochondrial dysfunction, endoplasmic reticulum stress, and neuronal apoptosis (Aluise et al. 2010; Kuzu et al. 2018). In the Tangpong et al study (2006) DOX autofluorescence was detected in the areas of the brain located outside the BBB, but strong TNF-α immunoreactivity was detected in the cortex and HP of DOX-treated mice.

Mitochondria adjust calcium absorption and redox signaling under physiological conditions (Zorov et al. 2014). In Park et al. study it was found that DOX is able to damage mitochondrial function in the HP, resulting in elevated mitochondrial ROS levels (Park et al. 2018). Moreover, glucose metabolism was decreased in both the HP and cortex bilaterally after i.t. injection of DOX. This damage to mitochondrial function is probably generated by the opening of the mitochondrial permeability transition pore (mPTP), which is assembled between mitochondrial membranes by three protein subunits, including cyclophilin D (CyP-D), an adenine nucleotide translocator (ANT) and VDAC. ROS initiates the activation of glycogen synthase kinase-3, which phosphorylates CyP-D into its active form. In addition, calcium dysregulation contributes to mitochondrial membrane depolarization and ANT conformational changes (Javadov et al. 2013).

On the other hand, Tangpong and co-workers proved that injection of the antibodies against TNF-a or iNOS prevented the damage of mitochondrial oxidative reaction in mice. This suggests that DOX reduces mitochondrial function via inflammatory reaction and NO• production (Tangpong et al. 2007).

It was also found that DOX can damage the progenitor neuronal degradation pathways and neuronal lysosomes. Moreover, it is able to up-regulate autophagy, and affect the clearance of the autophagic marker p62 protein (Tangpong et al. 2007). Moruno-Manchon and co-workers showed that DOX impairs neuronal autophagy in cortices from rat embryos (E19), leading to the accumulation of p62. The brains obtained from mice exposed to the action of pegylated liposomal DOX exhibited autophagosomes, lipofuscin, and lipid droplets. Interestingly, the authors' results also showed that lysosomes were less acidic in neurons treated with DOX. Furthermore, elevated levels of the transcription factor EB (TFEB), increased the survival of DOX‐treated neurons. Moreover, 2‐Hydroxypropyl‐β‐cyclodextrin (HPβCD), TFEB activator, also promoted neuronal survival (Moruno-Manchon et al. 2016).

## Increasing the Therapeutic Effect of DOX / Reduction of DOX Neurotoxicity

There have been many attempts in reducing potential DOX side effects including neurotoxicity, while simultaneously increasing the therapeutic efficacy by using various targeted delivery approaches. Nanotechnology is a promising tool to overcome cancer therapy limitations, including the systemic side effects, and to increase the therapeutic effectiveness of those drugs. A number of nanomaterials have been applied for this purpose e.g., polymeric nanoparticles, liposomes, dendrimers, metallic nanoparticles, or hydrogels.

Pegylated liposomal doxorubicin (PLDOX) is a preparation of DOX in which the molecule itself is delivered in vesicles called liposomes made of various lipids with an outer shell of polyethylene glycol. This procedure allows to release a higher dose of DOX within the tumor tissues, and a lower dose in normal tissue (Gordon et al. 2000). Despite the fact that PLDOX is associated with a number of side effects, cardiotoxicity has been shown to be extremely rare, compared to free DOX. Moreover, some data indicate that PLDOX may be much safer for the CNS than DOX and other anthracyclines. Cianfrocca et al. evaluated the efficacy of PLDOX among 85 women with platinum-resistant ovarian cancer who had previously received up to three courses of chemotherapy with PLDOX. Overall social well-being and cognitive functioning were found to improve with treatment (Cianfrocca et al. 2010). Contrary to this data, in a study by Crivellari and co-workers, the patients on PLDOX reported worse cognitive and physical functioning compared to non-PLDOX patients’ regimens (mostly cyclophosphamide+ methotrexate) (Crivellari et al. 2013). It should be noted, however, that these studies were not compared to free DOX.

Hydrogels are another class of nanomaterials used as carriers. They can be modified to reversibly bind multiple drug molecules onto a polymer molecule and have been widely studied as drug carriers for biomedical applications. Their advantages include reducing the toxicity of the free drug, decreasing the amount of drug needed by increasing circulation time, and promoting solubilization of hydrophobic drugs. For example, Dadsetan and co-workers (2010) used oligo(poly(ethylene glycol) fumarate) (OPF) hydrogels modified with small negatively charged molecules, sodium methacrylate (SMA), for delivery of DOX (Dadsetan et al. 2010) for cancer treatment in a human osteosarcoma cell line. The results showed that DOX released from the charged hydrogels remained biologically active and able to kill cancer cells.

Another carrier for DOX is nanoparticles. For example, in the study of Norouzi et al. (2020) magnetic iron oxide nanoparticles (IONPs) stabilized with trimethoxysilylpropyl-ethylenediamine triacetic acid (EDT) were provided as a carrier of DOX for glioblastoma multiforme therapy (Norouzi et al. 2020). It was found that the DOX was released from the DOX-EDT-IONPs within 4 days. The DOX-EDT-IONPs proved to be effective in the induction of cell death, proliferation inhibition, and ROS generation in glioma cell line U251. Overall, the developed approach allows the chemotherapeutic to defeat both the BBB and glioma cells with multidrug resistance while providing site-specific magnetic targeting (Norouzi et al. 2020).

However, an increase of DOX concentration in the brain, caused by the carriers, can lead to neurotoxic effects. In the review of Teleanu et al., attention was drawn to the need to assess the potential toxicity of nanomaterials. The authors of the study claim that there are several available toxicity evaluations, including nanomaterial characterization in both vitro and in vivo models, but also underline the lack of standardized and reliable neurotoxicological studies (Teleanu et al. 2019). The key finding here is that each type of nanomaterial from which the carriers are made exhibits at least minimal levels of neurotoxicity. Meticulous evaluation is therefore crucial to designing safer systems and reducing their side effects. To reduce neurotoxicity, it is necessary to remove toxic materials from the composition of carriers, shorten the exposure period, and control their size and shape (Teleanu et al. 2019).

Currently, it is also being investigated whether various experimental drugs and those used in the clinic can abolish DOX-induced neurotoxicity.

The data of Jantas and co-workers indicated that mGluR II/III activators could be neuroprotective against cell death induced by DOX, although that effect could differ dependent on the undifferentiation state of SH-SY5Y cells and the model of cellular injury (Jantas et al. 2015). AZ12216052 (a positive allosteric modulator at mGlu8 receptors), as well as UBP1112 (a group III mGluR antagonist) partially inhibited the cell damage induced by DOX in undifferentiated -SH-SY5Y cells (Jantas et al. 2018). The other studies of the same group showed that KU-55933 (ATM Kinase Inhibitor) inhibited the cell death induced by DOX in undifferentiated and retinoic acid-differentiated -SH-SY5Y cells, with a more pronounced effect in the latter cell phenotype (Chwastek et al. 2017). Memantine, an uncompetitive NMDA receptor antagonist was also studied as an agent that may reduce DOX neurotoxicity. Jantas and Lasoń (2009) in their study on the protective effects of memantine against DOX neurotoxicity in primary neuronal cell cultures suggest that this protective effect of memantine seems not to be dependent on caspase-3 activity and the antagonistic action on NMDA receptor (Jantas and Lasoń 2009). Furthermore, other data of Jantas et al. (2014) showed the predominant neuroprotective effect of tianeptine, was connected with attenuation of DNA fragmentation evoked by DOX cell damage in primary neurons and in retinoic acid-differentiated -SH-SY5Y cells (Jantas et al. 2014).

Jantas and co-workers (2018b) showed also protective effects of the mGluR7 allosteric agonist AMN082 against various harmful stimuli in glia, neuronal and neuronal-glia cell cultures. The group demonstrated that AMN082 with similar efficiency suppressed the glia cell damage evoked by staurosporine and DOX. The AMN082-mediated glioprotection was mGluR7-subsidiary and associated with decreased DNA fragmentation not involved in caspase-3 inhibition. Furthermore, it was found that the inhibitors of PI3single bondK/Akt and MAPK/ERK1/2 pathways blocked the protective effect of AMN082 (Jantas et al. 2018b).

In Sardi et al., studies it was found that morphine, dexamethasone, and ondansetron inhibit multidrug resistance proteins which are located on BBB, neurons, and glial cells, increasing the access of DOX to the rodent brain by efflux transporters competition (Sardi et al. 2013). The results showed that the level of DOX was significantly higher in all brain regions of rats pretreated with morphine or ondansetron in comparison to control tissues (Sardi et al. 2013).

Several in vitro studies have also demonstrated that BDNF and the insulin-like growth factor 1 (IGF-1) reduced DOX neurotoxicity in SH-SY5Y cells (Gil-Ad et al. 1999; Middlemas et al. 1999; Shavali et al. 2003).

El-Agamy and co-workers (2019) in their review paper gave also other examples of drugs that can reduce the risk of DOX-induced neurotoxicity. Selective serotonin reuptake inhibitor (SSRI) fluoxetine precluded chemotherapy-induced cognitive dysfunction in several animal models as evidenced by the improved performance of animals within NOR and T-maze tests. However, giving this example, it should be noted that SSRI during chemotherapy may not be beneficial because of the adverse effects experienced by a large subset of the subjects that may have sometimes led to termination of the study (El-Agamy et al. 2019). El-Agamy also believes that antioxidant treatment would be a promising strategy for combating chemotherapeutic-induced cognitive impairment, as it mainly focuses on blocking neurotoxic pathways. Several preclinical studies have shown that antioxidant treatment precluded chemotherapy-induced oxidative stress and cognitive impairment. For example, a xanthone derivative from garcinia mangosteens prevented DOX-mediated oxidative stress in the brain as evidenced by diminished production of protein carbonyl, nitrotyrosine and 4-hydroxy-2′-nonenal-adduced proteins in brain tissue (El-Agamy et al. 2019).

## Conclusions

The side effects of cancer pharmacotherapy are constantly a huge medical challenge.

Still, the most attention is paid to the mechanisms of side effects in the field of the cardiovascular system. Given that cancer patients often suffer from depressive disorders, more attention should be paid to the effects of medications that can exacerbate its symptoms, including cognitive decline. The effects of DOX leading to neurotoxicity is still not well understood. Other studies in vitro and in vivo are needed to investigate the mechanism of the neurotoxic action of DOX in the nervous system. Thus, identify the potential methods for protecting the CNS against the effects of DOX, widely used as a chemotherapeutic agent in the treatment of various types of cancer. In light of the information summarized above, it seems that research into various experimental drugs and those used in the clinic that can potentially abolish DOX-induced neurotoxicity is highly relevant.

## Data Availability

Not applicable
